# The Effect of Bacterial Peptide p28 on Viability and Apoptosis Status of P53-null HeLa Cells

**DOI:** 10.15171/apb.2019.078

**Published:** 2019-10-24

**Authors:** Haniyeh Abuei, Abbas Behzad-Behbahani, Fatemeh Faghihi, Ali Farhadi, Gholam Reza Rafiei Dehbidi, Mohammad Pirouzfar, Farahnaz Zare

**Affiliations:** ^1^Department of Medical Laboratory Sciences, School of Paramedical Sciences, Shiraz University of Medical Sciences, Shiraz, Iran.; ^2^Diagnostic Laboratory Sciences and Technology Research Center, School of Paramedical Sciences, Shiraz University of Medical Sciences, Shiraz, Iran.; ^3^Department of Oral and Maxillofacial Pathology, School of Dentistry, Shiraz University of Medical Sciences, Shiraz, Iran.

**Keywords:** p28, p53, Null HeLa cells, Apoptosis, Viability

## Abstract

***Purpose:*** Despite all the efforts for discovery of efficient anti-cancer therapeutics, cancer is still a major health concern worldwide. p28 is a bacterial small peptide which has been widely investigated due to its preferential cell internalization and anti-cancer activities. Intracellularly, p28 offers its anti-cancer traits by impeding the degradation of tumor-suppressor protein "p53". In this study, we investigated the potency of p28 in inducing apoptosis or decreasing cell viability in p53-null "HeLa" cell line.

***Methods:*** The coding sequence for p28 peptide was obtained from Pseudomonas aeruginosa by PCR amplification of the p28 gene. The coding gene was cloned in pET-28a vector and transformed into E. coli bacterial host. Subsequently, the expressed peptide was purified using Ni-NTA chromatography system and introduced into the target cells. The anti-proliferative and apoptotic activity of p28 on HeLa and HEK-293 cells were investigated using MTT and PEAnnexin V Flowcytometry assays.

***Results:*** Sodium dodecyl sulfate-polyacrylamide gel electrophoresis (SDS-PAGE) and Western blotting confirmed the expression of p28 peptide in the bacterial host. Bradford assay revealed a concentration of 0.05 mg/mL for the purified p28. MTT assay of cells treated with p28 at concentrations of 0, 0.5, 1, 2 and 2.5 µM indicated 24h viability values of 97%, 89%, 88%, 87% and 84% for HeLa cells, respectively. Data obtained from flowcytometry analyses revealed 24h apoptosis rate of 7.17%, 8.05%, 8.63% and 8.84% for HeLa cells treated with 0, 0.5, 1, and 2 µM p28, respectively.

*** Conclusion:*** MTT and flowcytometry apoptosis assays suggest no statistically significant effect of p28 on the viability and apoptosis status of p53-null HeLa cells when results compared to data obtained from HEK-293 cells (P>0.05). These results imply that anti-cancer efficacy of p28 is directly dependent on the presence of p53, suggesting p28 as an inappropriate therapeutic agent for treatment of cancers with negative p53 status.

## Introduction

Cancer, defined as the malignant and deregulated ‎growth of cells, is still a major cause of death in the 21st century.^1^ According to GLOBOCAN estimation, more than 18 million people were diagnosed with various types of cancer worldwide and nearly 10 million died because of cancer in 2018. It is also estimated that until 2030 there will be 30 million cancer deaths each year.^2,3^ The underlying deriver mechanism behind the cancer malignancies involves a sequence of molecular events such as mutations ‎and dysregulation in the expression of many genes essential for regulating proliferation, ‎survival, and growth activities of a normal cells.^4,5^

Of all the dysregulations that contribute to cancer progression, p53 mutation is the most frequent alteration with the greatest impact on the development of human cancers, and its inactivity is a universal phenomenon observed in more than half of the human cancers.^6^ p53 has a pivotal role in inducing cell growth arrest, senescence, and apoptosis,^7^ thus, restoration of its activity seems undeniably to be an important step in preventing every kind of neoplasia and cancers^8,9^ making it a suitable target for discovery of novel cancer treatment strategies. Currently, there are several cell lines available with various p53 status including wild-type, mutated, or null cancer cell models each of which are suitable for different study designs.^10-13^

In the last decade, a novel anti-cancer therapeutic agent known as p28 has attracted much attention.^14,15^ p28 is a small cell penetrating peptide derived from Azurin; a redox protein found in *Pseudomonas aeruginosa.*^16,17^ This peptide can penetrate into cancerous cells preferentially and exert anti-proliferative and anti-angiogenesis effects on the malignant tissue.^18-20^‎

Upon entering the target cell, p28 forms a complex with p53 either in its wild-type or mutated form and prevents it from binding to the E3 ubiquitin ligase Cop1 which ultimately leads to an elevation in the cellular concentration of p53.^21-23^ Consequently, p21 and p27 will be upregulated while the formation of CDK2–cyclin A mitotic complex and production of FoxM1 -a transcription factor essential for cell cycle progression will be hindered.^24,25^ Taken together, it appears that p28 affects cancerous cells mainly in a p53-mediated manner. Nevertheless, in a number of cancers such as human papillomavirus (HPV) related cervical carcinoma, despite no DNA mutations in p53 gene, its protein is not present inside the cell,^26,27^ which would be an obstacle for p28 to apply its anticancer activities.

In this study, we investigated whether the recombinant p28 can alter the viability or apoptosis level of HeLa cell a p53-null^26,28^ cervical cancer cell line. Additionally, we used p53-wild type HEK-293 cells as normal non-cancerous cells.

## Materials and Methods

### Cell Culture

HeLa, MCF-7 (p53 wild type cancerous cells as a positive control), and HEK-293 cells (purchased from Pasteur Institute of Iran, Tehran, Iran) were cultured in Dulbecco’s Modified Eagle Medium (DMEM) supplemented with 10% fetal bovine serum (Gibco, Thermo Fisher Scientific, Massachusetts, USA) and incubated at 37°C and 5% CO2.

### Polymerase Chain Reaction Amplification of p28 Gene

Total DNA was isolated from *P. aeruginosa* bacterial strain (gifted from Department of Medical Laboratory Sciences of Shiraz University of Medical Sciences (SUMS), Shiraz, Iran) using boiling method as described by other studies^29,30^ and used as a template for polymerase chain reaction**(**PCR) amplification of p28 gene. p28-Forward and -Reverse primers (Table 1) with appropriate restriction sites for NdeI and BamHI restriction enzymes (Thermo Scientific, Massachusetts, USA)‎ were designed using Allele Id version 7.5 (Premier Biosoft, California, USA) and ordered for synthesis (Metabion, Steinkirchen, Germany). PCR was carried out in a 20 µL reaction mixture containing Hot-start Taq polymerase (Ampliqon, Odense, Denmark).PCR cycles consisted of15 minutes of initial denaturation at 95°C, 30 seconds of denaturation at 95°C, 30 seconds of annealing at 58°C, and 30 seconds of elongation at 72°C in addition to a final extension for 5 minutes at 72°C.

**Table 1:** Primers used for amplification of p28

**Primer**	**Sequence**
p28-Forward	5'-ATTGGATCCATGCTGAGCACCGCCG-3'
p28-Reverse	5'-‎TACAAGCTTTAGGTCGTCGGGCTT-3'

### Expression and purification of recombinant p28

The 84 bp fragment of p28 gene was cloned into pET-28a expression plasmid vector (provided by Diagnostic Laboratory Sciences and Technology Research Center, Shiraz, Iran) using NdeI and BamHI restriction enzymes and T4 DNA ligase (Fermentas, Vilnius, Litvanya). The constructed vector was authenticated by enzyme digestion and Sanger sequencing. The plasmid vector was transformed into *E. coli* BL21 (DE3) bacteria (gifted from Department of Medical Laboratory Sciences of SUMS, Shiraz, Iran) and cultured on agar plate containing kanamycin (70 µg/mL). A single colony of the transformed bacteria was inoculated into 50 mL of LB broth (70 µg/mL of kanamycin), incubated at 37°C until the OD 600 absorbance reached 0.5–0.6. Isopropyl β-D-1-thiogalactopyranoside (IPTG; Sigma-Aldrich, St. Louis, Missouri, USA) at a final concentration of 1 mM was used to induce the expression of the heterologous protein. After four hours of incubation, bacteria were centrifuged and lysed by sonication (6×30 seconds, followed by 30 seconds cooling intervals) in lysis buffer (50 mM NaH_2_PO_4_, 500 mM NaCl, 10 mM imidazole and 8 M urea, pH 8). The lysis solution was centrifuged at 10 000 g for 30 min at 4°C, the supernatant fractions were loaded on Ni-NTA columns (Qiagen, Hilden, Germany), and the protein of interest was purified by affinity chromatography method. The column was washed with Native Washing Buffer (50 mM NaH_2_PO_4_, 500 mM NaCl, 50 mM imidazole and 8 M ‎urea, pH 8), and finally the 6×His tagged p28 peptide was eluted using Native Elution Buffer (50 mM NaH_2_PO_4_, 500 mM NaCl, 250 mM imidazole and 8M urea, pH 8). The eluted p28 solutions were used for dialysis to withdraw the urea.

### Protein assays

Twelve percent sodium dodecyl sulfate-polyacrylamide gel electrophoresis (SDS-PAGE) and Bradford assay were used to evaluate the protein expression and purified fractions. Moreover, Western Blot analysis using polyvinylidene difluoride (Merck, Darmstadt, Germany) and horseradish peroxidase (HRP) -conjugated anti-His tag antibodies (Abcam, Cambridge, United Kingdom) were used to approve the purification of p28. Briefly, samples of eluted fractions were separated by 12% SDS-PAGE and transferred onto a PVDF membrane. The membrane was immersed in the blocking buffer and then it was washed with Tris-buffered saline-Tween 20 (TBST) buffer. Then, the membrane was incubated in the HRP-conjugated anti His-tag antibody solution for 90 minutes at room temperature. After incubation, the membrane was washed again with TBST buffer, and protein was exposed to 3, 3-Diaminobenzidine solution (Sigma-Aldrich, St. Louis, Missouri, United States).

### Cell viability assay

The cytotoxic effect of the purified recombinant p28 peptide on the viability of HeLa, HEK 293 and MCF7 cells was evaluated using MTT assay. Initially, cells were seeded into 96-well plates at the density of 8000 cells/well and incubated for growth. Afterwards, the cells were treated with different concentrations of p28 peptide (0.5, 1, 2 and 2.5 µM) and incubated for 24 hours. Then, 20 µL of 3-(4, 5-dimethylthiazol-2-yl)-2,5-diphenyltetrazolium bromide (Sigma-Aldrich, St. Louis, Missouri, United States) at a concentration of 5 mg/mL was added to each well. Finally, after 4 hours of incubation, 100 µL of Dimethyl sulfoxide (Sigma-Aldrich; St. Louis, Missouri, United States) was added onto the wells and the absorbance was measured at 570 nm wavelength using an ELISA reader instrument (Tecan Infinite M200, Männedorf, Switzerland).‎

### Cell apoptosis assay

Once HeLa and HEK 293 cells reached the conﬂuency of 70%, they were treated with recombinant p28 peptide at the concentrations of 0.5, 1, and 2 µM. After 24 hours, the apoptosis level in the treated cells was evaluated using PE-AnnexinV/7AAD kit (BD Biosciences; San Jose, California, USA) according to the manufacturer’s instructions.‎

### Statistical analysis

GraphPad Prism 8.0 (GraphPad Software, Inc.) was used for all analyses. The MTT and apoptosis assays were performed in three independent experiments. One-way ANOVA followed by Dunnet’s post-hoc test were the method of choice for the statistical assessments.

## Results and Discussion

### PCR amplification of p28 gene

**Figure 1:**
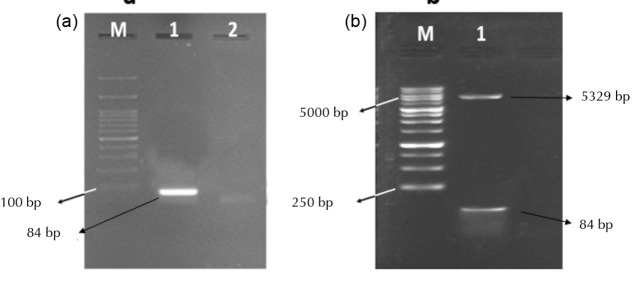
Gel electrophoresis associated with p28 gene. (a) PCR amplification of p28 gene. Lane1: DNA extracts of *P. aeruginosa* was used as PCR template. Lane 2: non-Template Control (NTC). M: 100 bp DNA ladder (b) Enzymatic digestion of p28-pET28a plasmid construct using BamHI and NdeI restriction enzymes. Lane 1: The recombinant p28-pET28a vector after digestion. M: 250-10000 bp DNA ladder.

p28 gene was PCR amplified by using DNA extracted from *P. aeruginosa*. From each of forward and reverse primers, 10 pmoles was added to a mixture containing Hot-start Taq DNA polymerase. Once the PCR was performed, an 84 bp band was visualized on the 2% agarose gel electrophoresis (Figure 1a).

In this study, the amplification was performed by employing a PCR Mastermix containing Hot-start Taq polymerase enzyme. Hot-start Taq polymerase was engineered to stay idle during setup mixing procedures, thus non-specific amplification did not happen. However, after an appropriate incubation at the optimal activation temperature (i.e. 95°C for 15 min), the enzyme became activated. Ultimately, the amplification was performed with a higher specificity and a greater yield.

### Validation of construction of p28-pET28a vector

After cloning, pET28a plasmids were subjected to restriction digestion by BamHI and NdeI restriction enzymes in order to determine whether the 84b p28 insert is cloned successfully (Figure 1b). Moreover, DNA Sanger sequencing further confirmed the success of the cloning procedure.

### Expression and purification of recombinant p28 peptide

**Figure 2:**
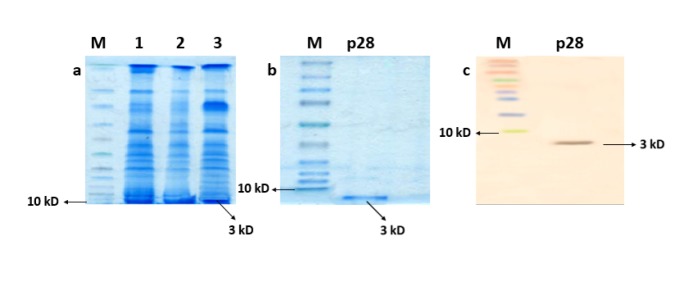
(a) SDS PAGE analysis of p28 peptide expression. M: Size marker (10-180KD). Lane 1: Protein expression status in untransformed *E. coli* bacteria. Lane 2: Protein expression prior to induction with IPTG. Lane 3: p28 peptide expression after induction of transformed bacteria with 1mM IPTG. The 3 kD band indicates overexpression of P28 peptide. (b)‎ Purification of p28 peptide (3 kD) using Ni-NTA Purification ‎System. (c) Western blotting approved the p28 overexpression with 3 kD molecular weight.‎

After 4 hours of IPTG induction, the expression of p28 was confirmed using SDS-PAGE assays indicated in Figures 2a and 2b, and an intensified band at 3 kD confirms the overexpression of p28 peptide in the induced transformed *E. coli.* Furthermore, Bradford assay quantification of the obtained peptide solutions revealed a concentration of 0.05 mg/mL for the purified p28. The results indicated that p28 was not expressed in untransformed or uninduced *E. coli* BL21 host, suggesting that the cloning, extraction, and purification of p28 were performed successfully. The purification of p28 peptide was further confirmed by western blotting assays (Figure 2c).

### MTT assay

**Figure 3:**
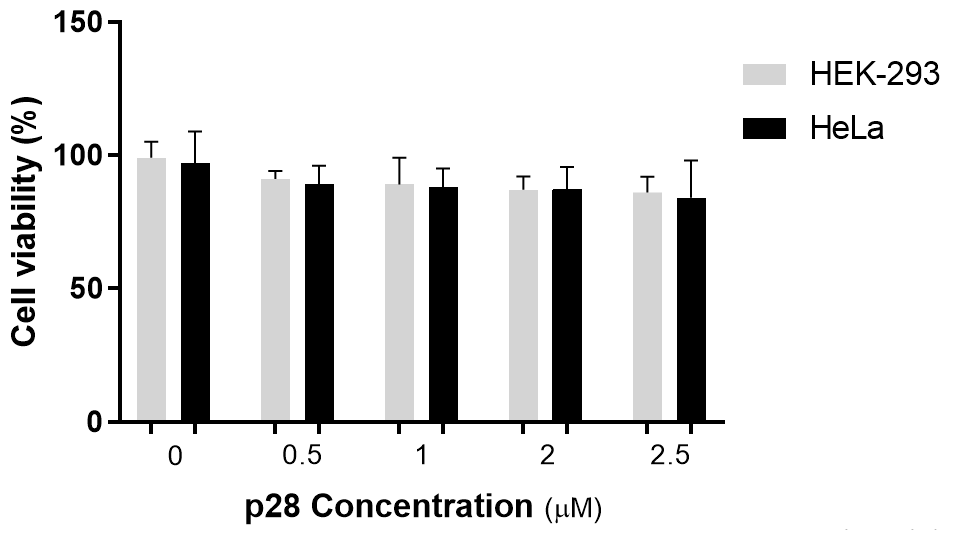
The effect of p28 on the viability of HeLa and HEK-293 cell. Cells were treated with p28 at concentrations of 0.5, 1, 2 and 2.5 µM, and their viability was evaluated by MTT assay after 24 hours. The results show no statisticaly significant reduction in the viability of treated HeLa and HEK-293 cells (*P* > 0.05). (Statistical analysis was performed using one-way ANOVA followed by Dunnet’s post-hoc test).‎

MTT assay was used for the determination of the cytotoxic effects of the purified p28 *in vitro*. p53-null HeLa cancerous cells, MCF7 cells and HEK-293 normal cells were treated with p28 peptide in various concentrations (0.5, 1, 2 and 2.5 µM) for 24 hours. The results of MTT assay are shown in Figure 3 (data is not shown for MCF7 cells). Incubation of the HEK-293 cells with any of p28 concentrations led to no significant changes in the measured absorbance. Similarly, test groups of HeLa cells exposed to various concentrations of p28 did not show any significant decrease in their MTT assay measurements. These results suggest that p28 peptide had no significant anti-proliferative effects on either HeLa or HEK-293 cells (*P* > 0.05).

### Apoptosis assay

In order to further determine the effect of p28 peptide, as a pro-apoptotic agent, on the HeLa and HEK-293 cells, AnnexinV/7-AAD ﬂow cytometry assay was performed. Test groups of HeLa and HEK-293 cells were treated with p28 peptide at a series of concentrations (0.5, 1, 2 and 2.5 µM) and incubated for 24 hours. Afterwards, the test and control groups were analysed using a flowcytometry cell analyser.

**Figure 4:**
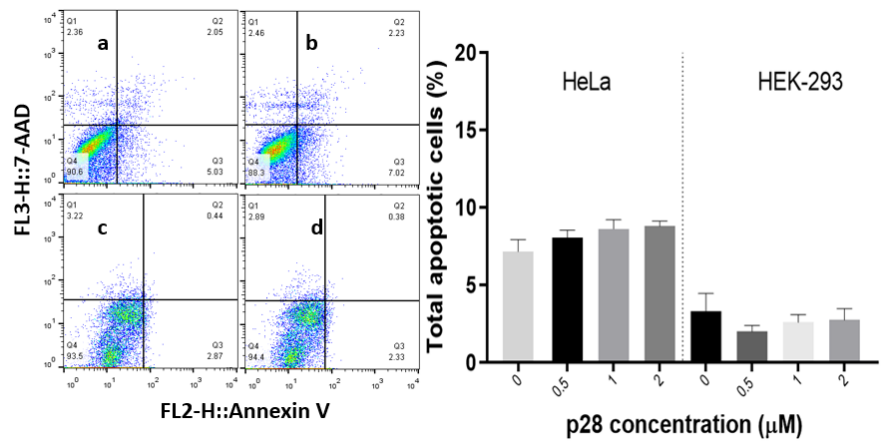
The measurement of apoptosis of HeLa and HEK-293 cell treated with p28. Cells were treated with p28 at concentrations of 0.5, 1, and 2 µM, and their apoptosis level was evaluated by PE-Annexin V flowcytometry assay after 24 hours. The results were interpreted by FlowJo version 10.0 (FlowJo LLC). Dot plots a and b demonstrate apoptosis in untreated control group of HeLa cells and HeLa cells treated with 2 µM p28, respectively, while plots c and d are associated with untreated control group of HEK-293 cells and HEK-293 groups treated with 2 µM p28, respectively. The results show no statistically significant changes in apoptosis of HeLa or HEK-293 cells (*P* > 0.5). (Statistical analysis was performed using one-way ANOVA followed by Dunnet’s post hoc test)‎.

The results (demonstrated in Figure 4) suggest that treating both HeLa and HEK-293 cells with p28 peptide had no significant effect on their apoptosis level compared to untreated control cells (*P* > 0.05). This finding confirmed that p28 would not affect apoptosis in non-cancerous cells or in cells with absent p53 status which suggests that p53-mediated mechanism of action of p28 as pro-apoptotic therapeutic agent.

p28 is a small peptide which has the ability to specifically penetrate cancer cells to induce apoptotic and anti-proliferative effects on them.^16,18^ To date, the knowledge on p28 suggests its p53-dependent action described as forming a complex with p53 by bounding to its DNA binding domain (DBD) and hindering its degradation;^21^ a process which ultimately contributes to induction of apoptosis in cancerous cells and preventing them from abnormal growth.^14^

In this study, we questioned whether p28 can affect the viability status of cancerous cells in the absence of p53. We designed a bacterial expression setting to produce recombinant p28 peptide in *E. coli* BL21 bacterial host, and purified it by using Ni-NTA chromatography system. Afterwards, we introduced the purified peptide to two different mammalian cell lines: HeLa, a cervical adenocarcinoma cell line in which p53 becomes degraded rapidly, and HEK-293, a non-cancerous human kidney cell with normal p53 status.^31^ We evaluated the anti-proliferative activity of p28 using MTT assay. We also ‏measured apoptosis in treated cells to determine whether p28 affects the apoptosis level of our target cells. Taken together, p28 had no significant effects on the viability and apoptosis level of both HeLa and HEK-293 cells which was in consistence with the findings of previous studies that suggest p53-dependency of p28. In 2009, Yamada et al reported that p28 may induce apoptosis in MCF-7 cells which is a p53-wild type breast cancer cell line but not in breast cancer p53 dominant-negative MDD2 cells.^25^ Another study in 2013 suggested p28 had a significant activity in cells expressing wild-type p53 or in those with mutations in regions other than DBD, while no p28 activity was observed in cells with p53 DBD mutations including TE-85 osteosarcoma, RD rhabdomyosarcoma, HT-29 colon cancer, ES-2 ovarian cancer, and MIA-and Paca2 pancreatic cancer. Additionally, p28 showed no effectivity on MEL-6 melanoma cells which have null status for p53.^22^ In 2016, Yamada et al also demonstrated that p28 is not active against the p53 null cell line PC3 despite its significant anti-cancer efficacy on p53-wild-type cells.^18^

## Conclusion

p28 is a small peptide which has the ability to impede cell proliferation and induce apoptosis in cancer cells preferentially. However, the results of our study revealed that the anti-cancer efficacy of p28 is directly dependent on the presence of p53 suggesting its inapplicability as a therapeutic agent in cancers with null-p53 status such as HPV-related cervical cancers.

## Ethical Issues

The research project has been approved by the Ethics Committee of Shiraz University of Medical Sciences (IR.SUMS.REC.1396.S73(.

## Conflict of Interest

The authors declare no conflict of interest.

## Acknowledgments

The authors would like to thank Dr. AY Farahmandi at the Department of English Language, Shiraz University of Medical Sciences for his invaluable assistance in editing this manuscript. The project was financially supported by Shiraz University of Medical Sciences, Shiraz, Iran under the agreement no. 95-01-10-13718.
